# Annegret F. Hannawa, Günther Jonitz: New Horizons in Patient Safety: Understanding Communication: Case Studies for Physicians

**DOI:** 10.3205/zma001128

**Published:** 2017-11-15

**Authors:** Kai P. Schnabel

**Affiliations:** 1 Universität Bern, Institut für Medizinische Lehre, Abteilung für Unterricht und Medien, Bern, Switzerland

## Recension

Written by Annegret Hannawa along with contributing co-author Günther Jonitz, this book is for all those working in healthcare. The German version is a translation of the English title and culturally adapted for a German audience. It is a more detailed introduction to communicating “safely” with patients and reaching consensus with other healthcare professionals. This book not only addresses the growing problem of miscommunication, which according to recent studies has taken on alarming dimensions, but also outlines the required core competencies to ensure “safe communication” in healthcare.

This is emphasized in the introduction by Gerd Gigerenzer and Sir Liam Donaldson, who are very likely familiar to those dealing with patient safety.

The book is divided into two sections. The first one is dedicated to the principles of patient safety and interpersonal communication and presents a framework developed by the author which encompasses six core communication skills (SACCIA). The second section contains 39 critical incident descriptions organized according to level of care.

In the part covering the basics, Günther Jonitz persuasively argues the necessity of addressing this still very taboo subject in medicine.

Annegret Hannawa takes this up further in a descriptive section on communication and debunks various myths surrounding interpersonal communication. She explains the concept of “safe communication,” from the basic principles to her own approach (SACCIA->**S**ufficiency,** A**ccuracy, **C**larity, **C**ontextualisation, **I**nterpersonal **A**daptation) which enables an analysis of communication errors and the identification of five core competencies underpinning safe communication. The aim of this book is to reduce avoidable mistakes in the future.

The critical incident descriptions in the second part are based on real cases and range from near misses to seriously critical events. The main focus of the case examples is on inpatient care (28 cases), but there are also 11 outpatient cases to which Hannawa’s framework is applied and made easy to understand.

The book is an excellent reference work for case-based instruction and learning, particularly in interprofessional contexts. Together with the framework, the book offers an excellent teaching strategy in that it is possible to discover and discuss the concept and practice of safe communication step by step.

In aviation and in some areas of medicine (intensive medicine), this approach has already led to a clearer understanding that errors are less the result of an individual and more a collective event, something that can be made less probable in the future, if not ruled out, through institutional interventions.

It is our hope that the insights presented in this book also lead to a more serious consideration of the significance of communication in medical education, and that as a result not only the emotional relationship between doctor and patient improves, but that patient safety also increases.

## Competing interests

The author declares that he has no competing interests.

